# Genome-wide investigation of WRKY transcription factors in Tartary buckwheat (*Fagopyrum tataricum*) and their potential roles in regulating growth and development

**DOI:** 10.7717/peerj.8727

**Published:** 2020-03-05

**Authors:** Wenjun Sun, Zhaotang Ma, Hui Chen, Moyang Liu

**Affiliations:** 1 School of Agriculture and Biology, Shanghai Jiao Tong University, Shanghai, China; 2 College of Life Science, Sichuan Agricultural University, Ya’an, China

**Keywords:** *FtWRKY*, Tartary buckwheat genome, Fruit, Development

## Abstract

**Background:**

The WRKY gene family plays important roles in plant biological functions and has been identified in many plant species. With the publication of the Tartary buckwheat genome, the evolutionary characteristics of the WRKY gene family can be systematically explored and the functions of *Fagopyrum tataricum WRKY* (*FtWRKY*) genes in the growth and development of this plant also can be predicted.

**Methods:**

In this study, the *FtWRKY* genes were identified by the BLASTP method, and HMMER, SMART, Pfam and InterPro were used to determine whether the *FtWRKY* genes contained conserved domains. The phylogenetic trees including *FtWRKY* and *WRKY* genes in other plants were constructed by the neighbor-joining (NJ) and maximum likelihood (ML) methods. The intron and exon structures of the* FtWRKY* genes were analyzed by the gene structure display server, and the motif compositions were analyzed by MEME. Chromosome location information of *FtWRKY* genes was obtained with gff files and sequencing files, and visualized by Circos, and the collinear relationship was analyzed by Dual synteny plotter software. The expression levels of 26 *FtWRKY* genes from different groups in roots, leaves, flowers, stems and fruits at the green fruit, discoloration and initial maturity stage were measured through quantitative real-time polymerase chain reaction (qRT-PCR) analysis.

**Results:**

A total of 76 *FtWRKY* genes identified from the Tartary buckwheat genome were divided into three groups. *FtWRKY* genes in the same group had similar gene structures and motif compositions. Despite the lack of tandem-duplicated gene pairs, there were 23 pairs of segmental-duplicated gene pairs. The synteny gene pairs of *FtWRKY* genes and *Glycine max WRKY* genes were the most. *FtWRKY42* was highly expressed in roots and may perform similar functions as its homologous gene *AtWRKY75*, playing a role in lateral root and hairy root formation. *FtWRKY9*, *FtWRKY42* and *FtWRKY60* were highly expressed in fruits and may play an important role in fruit development.

**Conclusion:**

We have identified several candidate *FtWRKY* genes that may perform critical functions in the development of Tartary buckwheat root and fruit, which need be verified through further research. Our study provides useful information on *WRKY* genes in regulating growth and development and establishes a foundation for screening *WRKY* genes to improve Tartary buckwheat quality.

## Introduction

The WRKY transcription factor family acts a vital part during the growing and developing process of higher plants (Wei et al., 2012). The most basic characteristic of this family member is that it contains a WRKY conserved domain, which consists of approximately 60 amino acid residues. Some members of the WRKY family have one conserved domain, while others have two. The conserved domains include a conserved heptapeptide WRKYGQK at the N-terminus (this heptapeptide also appears as WRRYGQK, WSKYGQK, WKRYGQK, WVKYGQK and other forms) and a distinctive zinc-finger-like motif, C2H2 or C2HC at the C-terminus (Rushton et al., 2010; Zhen et al., 2005). The structural characteristics of WRKY TFs at the N-terminal and C-terminal determine their binding to W-box (TTGACC/T) or sugar-responsive cis-element (SURE) cis-acting elements in the specific gene promoters (Chuanxin et al., 2003; Ciolkowski et al., 2008; Rushton et al., 1995). In *Arabidopsis thaliana* (*A. thaliana*), 72 *Arabidopsis thaliana* WRKY (*AtWRKY*) members were grouped into Group I, II and III because of the difference in the amount of conserved domains and zinc finger structures (Sultan et al., 2016). The members in Group I have a WRKY conserved region at the C-terminal and N-terminal, respectively, and their zinc finger structure is C2-H2 (Eulgem et al., 2000). Group II has only one WRKY conserved region at the N-terminus, and the zinc finger structure is the same as that of Group I (Eulgem et al., 2000). Although Group III members only contain one WRKY conservative region, their zinc finger structure is C2-HC (Eulgem et al., 2000). According to the evolutionary relationship of the members from Group II, they can be grouped into five subfamilies, extending from IIa to IIe (Zhang & Wang, 2005).

Since the first cloning and identification of the *SPF1* from *Ipomoea batatas*, an increasing number of *WRKY* genes have been identified in other plants (Ishiguro & Nakamura, 1994). *WRKY* genes are involved in plant seed germination, plant branch root formation, regulation of plant flowering time, control of fruit maturation and leaf senescence, and these genes are also involved in the synthesis of plant secondary metabolites (Balazadeh, Riaã±O-Pachã3N & Mueller-Roeber, 2010; Devaiah, Karthikeyan & Raghothama, 2007; Gonzalez et al., 2016; Wei, Wang & Yu, 2016a; Zhou et al., 2016). The flowering process of plants is of great significance to the reproduction and evolution of plants (Yanjuan et al., 2014), and it is found that *AtWRKY71* can activate the expression of flowering genes and accelerate the flowering process of *A. thaliana* (Yu et al., 2016a). Leaf senescence may limit plant yield, and studies have shown that *AtWRKY6* can activate SIRK, an enzyme protein that is highly expressed in leaf senescence and plays an important role in leaf senescence (Silke & Somssich, 2002). *WRKY* genes not only play a role in plant development but also play an indispensable role in abiotic stress (Banerjee & Roychoudhury, 2015). Previous studies have shown that plant hormones, drought, salt, cold and other abiotic stresses can influence the expression of *WRKY* genes in rice and *A. thaliana* (Ramamoorthy et al., 2008; Seki et al., 2010; Singh, Foley & Onate-Sanchez, 2002). *WRKY* genes also participate in some signal transduction processes that are mediated through plant hormones (Phukan, Jeena & Shukla, 2016). *AtWRKY39* can promote the interaction between salicylic acid and jasmonate signaling pathways (Li et al., 2010). Despite the continuous evolution of plants, the *WRKY* genes have been relatively conserved, and it is precisely because of the continuous evolution and species diversity of plants that genes of the WRKY TF family can continue to expand (Rinerson et al., 2015). With the sequencing of increasing numbers of plant genomes, the WRKY gene families of different plants have been systematically and comprehensively analyzed, which establishes a foundation for us to study the evolutionary origin of WRKY TF family in great depth.

Widely cultivated Tartary buckwheat (*Fagopyrum tataricum* Gaertn.) includes 20 different types of medicinal and edible crop varieties and has considerable economic and nutritional value (Anton et al., 2012; Takanori, Kyoko & Ohmi, 2002). Tartary buckwheat fruits are rich in vitamin B, dietary fiber, protein and various minerals and are particularly high in niacin, magnesium, manganese and phosphorus (Bonafaccia, Marocchini & Kreft, 2003). Meanwhile, Tartary buckwheat fruit is rich in rutin and is useful in preventing liver damage, particularly inflammatory liver damage (Lee et al., 2013). The role of the *WRKY* genes has been systematically researched in many other species, such as *Cucumis sativus* L. (*C. sativus*) (Ling et al., 2011), *Vitis vinifera* L. (*V. vinifera*) (Wisła, 2014), A. thaliana (ülker & Somssich, 2004), *Oryza sativa* L.(*O. sativa*) (Ramamoorthy et al., 2008) and *Zea mays* L. (*Z. mays*) (Wei et al., 2012). He et al. (2019) identified 78 *FtWRKY* genes from Tartary buckwheat and simultaneously excavated *FtWRKY* genes involved in abiotic stress. However, the *FtWRKY* genes that regulate the growth and development of Tartary buckwheat have not been systematically identified. While this study mainly excavated the *FtWRKY* genes that regulate growth and development from the Tartary buckwheat genome. After identifying the FtWRKY gene family, we used SMART, Pfam and InterPro to determine whether the identified *FtWRKY* genes had conserved domains, and finally identified 76 *FtWRKY* genes from Tartary buckwheat genome. Although He et al. (2019) identified more genes, those redundant genes had no conserved domains. Therefore, this study has two goals. The first is to conduct a comparative analysis with the results of He et al. (2019)’s study. The second is to excavate the genes related to the regulation of growth and development by identifying the FtWRKY gene family. The gene structures, motif compositions, chromosome localizations, tandem duplications and segmental duplication events of 76 *FtWRKY* family members were thoroughly analyzed. In addition, we also explored the homologous gene pairs and evolutionary relations with *WRKY* genes in other plants, which was not found in the He et al. (2019)’s article. Many *AtWRKY* genes play important roles in growth and development (Devaiah, Karthikeyan & Raghothama, 2007; Silke & Somssich, 2002). To comprehensively explore the function of *FtWRKY* genes in growth and development, we selected a total of 26 *FtWRKY* genes homologous to *AtWRKY* from different groups and measured the expression levels of these genes in leaves, stems, roots, flowers and fruits at green fruit, discoloration and initial maturity stages. This study provides valuable clues regarding the specific functions of WRKY TF family members involved in Tartary buckwheat biological functions.

## Materials and Methods

### Materials

The Tartary buckwheat in our experiment grew in a greenhouse located in College of Life Sciences, Sichuan Agricultural University, China with 25 °C, 16 h of light and 8 h of darkness. We collected leaves, flowers, roots and stems from three mature Tartary buckwheat plants with similar growth. At the same time, we collected fruits at the green fruit stage (13 days after pollination, DAP), discoloration stage (19 days after pollination, DAP) and initial maturity stage (25 days after pollination, DAP), respectively. All samples were collected into a centrifuge tube, quickly placed in liquid nitrogen, and kept fresh at −80 °C.

### Gene identification and classification

In order to more accurately identify all *FtWRKY* genes with conserved domains from Tartary buckwheat genome, we carried out further screening based on the identification method reported by He et al. (2019). The candidate FtWRKY proteins identified based on HMMER 3.0 were BLASTp with all AtWRKY protein sequences downloaded from the TAIR library, and the identified FtWRKY proteins were confirmed to have a complete amino acid sequence. In addition to HMMER and SMART (Letunic, Doerks & Bork, 2012) used in He et al.’s article, we also used Pfam (Finn et al., 2014) and InterPro (Zdobnov & Apweiler, 2001) to verify whether the identified *FtWRKY* genes had WRKY core sequences, and the genes without WRKY core sequences were removed. In contrast to He et al.’s article, the *FtWRKY* genes identified above were again analyzed by BLASTp in NCBI to determine whether these genes belong to the WRKY gene family (He et al., 2019). Finally, 76 *FtWRKY* genes were identified. The sequence lengths, molecular weights (Mw), isoelectric points (PI) and subcellular localizations of these FtWRKY proteins are determined with the use of the ExPasy website (http://web.expasy.org/protparam/).

### Gene ontology (GO) annotation

To determine putative functions of the identified *FtWRKY* genes, GO analysis was performed using the Blast2GO gene ontology analysis tools (http://www.blast2go.com) to determine the GO annotation of all genes (Table S1) (Conesa et al., 2005).

### Phylogenetics, intron-exon structure, motif composition, cis-acting elements

In order to determine whether the grouping of 76 *FtWRKY* genes identified by us were consistent with the 78 *FtWRKY* genes identified by He et al. (2019), we constructed phylogenetic trees of *WRKY* genes in Tartary buckwheat and *A. thaliana* by NJ and ML methods by MEGA 7 (Kumar, Stecher & Tamura, 2016), respectively. Sequence alignment of all WRKY proteins is performed by MUSCLE (Edgar, 2004). The NJ method uses the Poisson model, while the ML method uses Jones-Tayler-Thornton (JTT) model. Different from the He et al.’s article, we also explored the evolutionary relationship of *WRKY* genes in multi species (He et al., 2019). WRKY proteins of *A. thaliana*, *Beta vulgaris* L.  (*B. vulgaris*), *Oryza sativa* L. (*O. sativa*), *Glycine max* (Linn.) Merr. (*G. max*), *Solanum lycopersicum* (*S. lycopersicum*), *V. vinifera* and *Helianthus annuus* L. (*H. annuus*) are obtained from the UniProt database (https://www.uniprot.org/). The phylogenetic tree including the abovementioned plants was also constructed through ML method by MEGA 7. Similarly, to determine whether the gene structure and motif composition of the 76 identified *FtWRKY* genes were consistent with those identified in He et al.’s article, we analyzed the exon/intron structure of the *FtWRKY* genes through a gene structure display server (GSDS: http://gsds.cbi.pku.edu.cn/) online program (Guo, Zhu & Xin, 2007), and analyzed the conservative motifs of recognized FtWRKY protein sequences with MEME program (http://meme-suite.org/tools/meme) and set the parameters to an optimum motif width of 6–200 and 10 motifs (Bailey et al., 2009). The amino acid sequences of every motif are in Table S2. We analyzed cis-acting elements of 2,000 bp upstream of all *FtWRKY* genes by PlantPAN software (http://plantpan.itps.ncku.edu.tw/), which were not mentioned in He et al.’s article (He et al., 2019).

### Distribution on chromosomes and duplication events of *FtWRKY* genes

The chromosome localization information of *FtWRKY* genes was obtained with gff files and sequencing files, and the obtained localization information was visualized by Circos (Krzywinski et al., 2009) to compare with the results of He et al.’s article (He et al., 2019). Gene duplication events were analyzed using default parameters in multiple collinear scan kits (MCScanx) (Wang et al., 2012). We defined tandem duplicated genes as homologous genes with more than 70% similarity within 200 kb on a single chromosome. Segmental duplications were defined as highly identical duplicated DNA fragments greater than 1 kb (Marques-Bonet, Girirajan & Eichler, 2009; Zhao et al., 2017). According to the above criteria, we identified the tandem duplicated genes and segmental duplicated genes in 76 *FtWRKY* genes, in order to compare with the results of He et al.’s (He et al., 2019). Different from He et al.’s article, we used Dual synteny plotter software (https://github.com/CJ-Chen/TBtools) (Chen et al., 2018) to explore the collinear relationship between the *FtWRKY* and *WRKY* genes from *A. thaliana*, *B. vulgaris*, *O. sativa*, *G. max*, *S. lycopersicum*, *V. vinifera* and *H. annuus*.

### Expression study of *FtWRKY* genes by qRT-PCR

Different from the focus of He et al.’s article, this study mainly identified the *FtWRKY* genes, which play an important role in the growth and development of Tartary buckwheat. Therefore, the expression levels of *FtWRKY* genes in leaves, flowers, stems, roots and fruits at three developing periods (13, 19 and 25 DAP) were detected by qRT-PCR. In Table S4, the primers for use were designed by Primer3 (http://frodo.wi.mit.edu/). With *FtH3* as an internal control, we use SYBR Premix Ex Taq II (TaKaRa) to perform qRT-PCR on a CFX96 Real-Time System (BioRad) (Li et al., 2019). Three biological replicates were employed in the experiment, and three technical replicates were employed in each biological replicate. The data were calculated with the 2^−(ΔΔ*Ct*)^ method, and the corresponding expression results were obtained (Livak & Schmittgen, 2001).

### Statistical analysis

We processed and analyzed our experimental data with the Origin Pro 2018b (OriginLab Corporation., Northampton, Massachusetts, USA), and we used the least significant difference (LSD) test to compare these data.

## Results

### Genome-wide identification of *FtWRKY* genes in Tartary buckwheat comparison with that of He et al.’s

**Figure 1:**
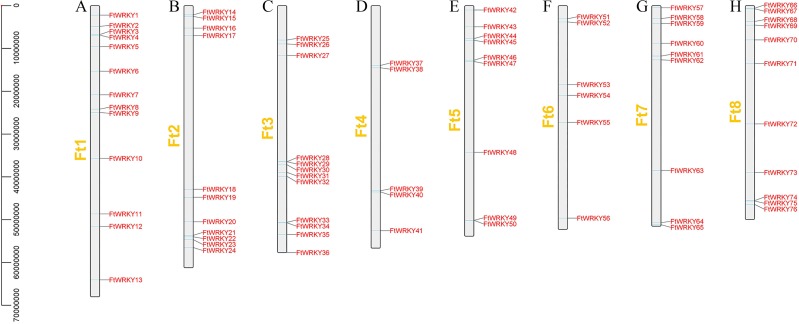
Schematic representation of the chromosomal distribution of tartary buckwheat *WRKY* genes. The chromosome number is indicated to the left of each chromosome. (A–H) stands for eight chromosomes of tartary buckwheat.

We identified 76 *FtWRKY* genes of Tartary buckwheat at the genomic level, but Ha et al. identified two more genes, including *FtPinG0006884300.01* and *FtPinG0001732900.01*. Through SMART, Pfam and InterPro identification, we found that *FtPinG0006884300.01* had no WRKY conservative domain, while *FtPinG0001732900.01* showed two alternative splicings of messenger RNA, we only retained the longest transcript. We also named them *FtWRKY1* to *FtWRKY76* according to their position on the chromosomes, but compared with He et al.’s article, the genes located on each chromosome are different (Fig. 1). *FtPinG0006884300.01* was located on chromosome 2 in He et al.’s article (He et al., 2019), and we have removed this gene. Therefore, compared with He et al.’s article, except for the same gene localization on chromosome 1, the gene localization on other chromosomes is different (Fig. 1). The molecular weight (Mw), isoelectric point of the 76 FtWRKY proteins were consistent with the reports of He et al. (Table S1). He et al.’s article predicted that all FtWRKY members were located in the nucleus, while we predicted that FtWRKY64 was located in the cytoplasm (Table S1). Meanwhile, the Blast2GO software was employed to confirm GO annotations in *FtWRKY* genes (Conesa et al., 2005) (Table S1). PseudoPipeand and PseuudoFinder were used to predict pseudogenes (Van Baren & Brent, 2006; Zhaolei et al., 2006), and no *FtWRKY* gene with pseudogene characteristics was found.

**Figure 2:**
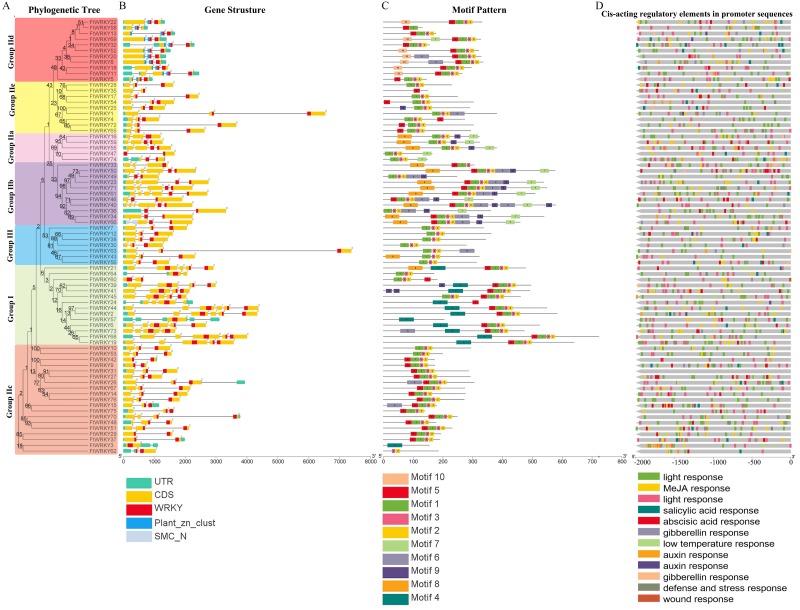
Phylogenetic relationships, gene structure, architecture of conserved protein motifs and cis-acting elements analysis of the *WRKY* genes from tartary buckwheat. (A) A phylogenetic tree based on the full-length sequences of tartary buckwheat WRKY proteins was constructed using Geneious R11 software. (B) Exon-intron structure of tartary buckwheat *WRKY* genes. Blue boxes indicate zinc finger structure; yellow boxes indicate coding regions; black lines indicate introns. The WRKY domains are indicated by red boxes. The numbers indicate the phases of corresponding introns. (C) Motif composition of tartary buckwheat WRKY proteins. The motifs (numbered 1–10) are displayed in differently colored boxes. The sequence information for each motif is provided in Table S2. The lengths of the proteins can be estimated using the scale at the bottom. (D) The cis-acting elements analysis of *FtWRKY* genes promoters. Blocks of different colors represent light responsiveness elements, low temperature responsiveness elements, salicylic acid responsiveness elements, abscisic acid responsiveness elements, MeJA responsiveness elements, auxin responsiveness elements, gibberellin responsiveness elements and defense, stress responsiveness elements and wound responsiveness elements.

We also analyzed the structure of *FtWRKY* genes, and the composition of the intron and the exon of *FtWRKY* genes was different compared with that of the He et al. All *FtWRKY* genes identified in He et al.’s article contain introns, and we identified *FtWRKY3* and *FtWRKY57* had no introns, other *FtWRKY* genes were interrupted by different numbers of introns, ranging from 1 to 5 (Fig. 2B). It can be also seen from Fig. 2B that each *FtWRKY* gene contained a WRKY conserved domain, and the members of Group I d contain a plant_zn_clust domain in addition to the WRKY conserved domain.

We used online MEME to analyze the motifs of 76 FtWRKY members and compare the results with those of He et al. (Fig. 2C). The motif length used in the He et al.’s article ranged from 15 to 49 bp (He et al., 2019), whereas the motif used for us ranged from 15 to 40 bp (Table S2). The motifs we chose were different from that of He et al., so the result of the motif composition of the *FtWRKY* genes was quite different. Each FtWRKY protein contained different motifs, but the motif composition of the same subgroup was relatively similar. Most proteins contain motifs 1, 2, and 3, but only FtWRKY3 in Group IIc and proteins of Group I contain the motif 4. Among all FtWRKY proteins, Group IIb members had the largest number of motif types (Fig. 2C). The similarity of motif constitution and structure of FtWRKY protein in the same group further supports the reliability of systematic evolution tree. Analysis of the cis-acting elements in their promoter region revealed that most *FtWRKY* genes contained light and phytohormone responsiveness elements, including MeJA, salicylic acid and abscisic acid responsiveness elements (Fig. 2D).

### Classification and evolutionary relationship analysis of the FtWRKY family comparison with that of He et al.’s

**Figure 3:**
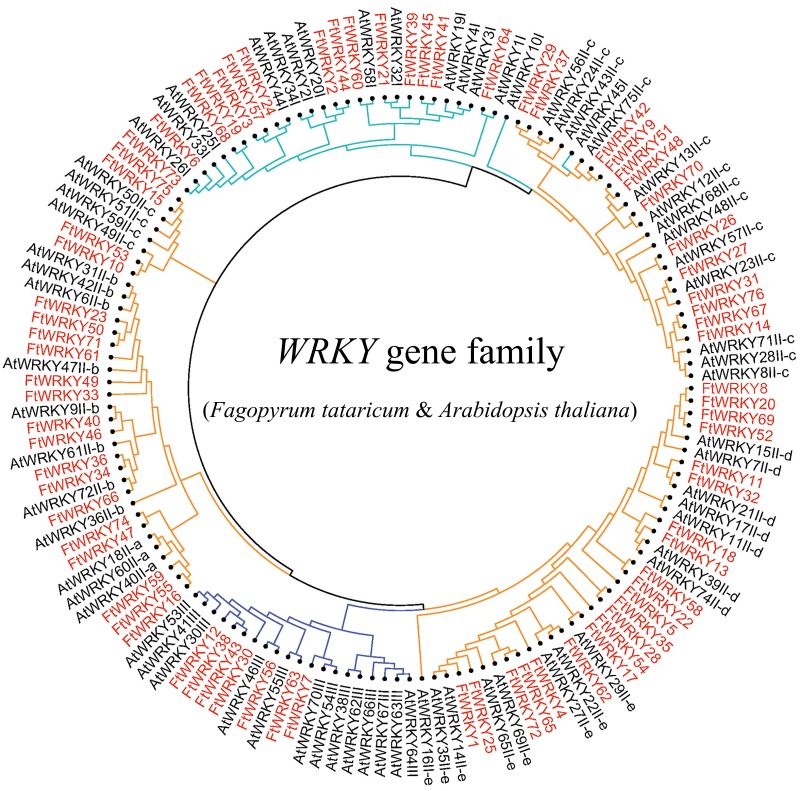
Unrooted phylogenetic tree representing relationships among the WRKY genes of tartary buckwheat and *A. thaliana* use ML method. The genes in tartary buckwheat are marked in red, while those in *A. thaliana* are marked in black. The different-colored arcs indicate different groups (or subgroups) of WRKY genes.

**Figure 4:**
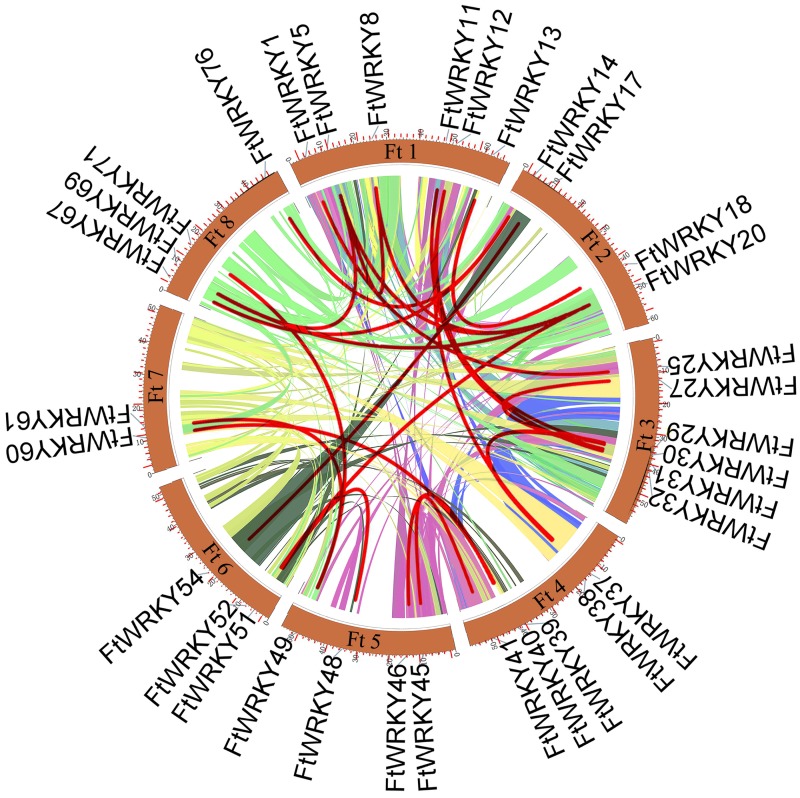
Schematic representations of the interchromosomal relationships of tartary buckwheat *FtWRKY* genes. The colored lines indicate the synteny blocks in the tartary buckwheat genome.

We conducted multiple sequence alignments between 72 AtWRKY proteins and 76 FtWRKY proteins and compare the results with those of He et al. (Fig. S2). We found that the WRKY domain of each protein was relatively conservative, and a total of 73 FtWRKY proteins had a highly conserved WRKYGQK sequence, while the WRKY domain of FtWRKY15, FtWRKY75 and FtWRKY49 had only a single amino acid change, which was consistent with the results of He et al. (2019) (Fig. S2). In order to compare with the grouping of *FtWRKY* genes in He et al.’s article, we constructed phylogenetic trees using ML and NJ methods. All the genes in the phylogenetic trees constructed by the two methods were divided into three groups, with each containing the same component of *FtWRKY* members (Fig. 3 and Fig. S2). The grouping of *FtWRKY* genes in the phylogenetic tree constructed by us was consistent with that reported by He et al. (Fig. 3 and Fig. S2). Segmental and tandem duplications are one of the reasons for the expansion of plant gene family (Cannon et al., 2004). Since our criteria for defining tandem duplication genes were different from those in He et al.’s article, we did not identify tandem duplication genes, whereas the He et al.’ s article identified two pairs of duplication genes (He et al., 2019) (Fig. 1). Although there are no tandem-duplicated genes, 23 pairs of segmental-duplicated genes may also promote the expansion of Tartary buckwheat WRKY family (Fig. 1 and Fig. 4).

**Figure 5:**
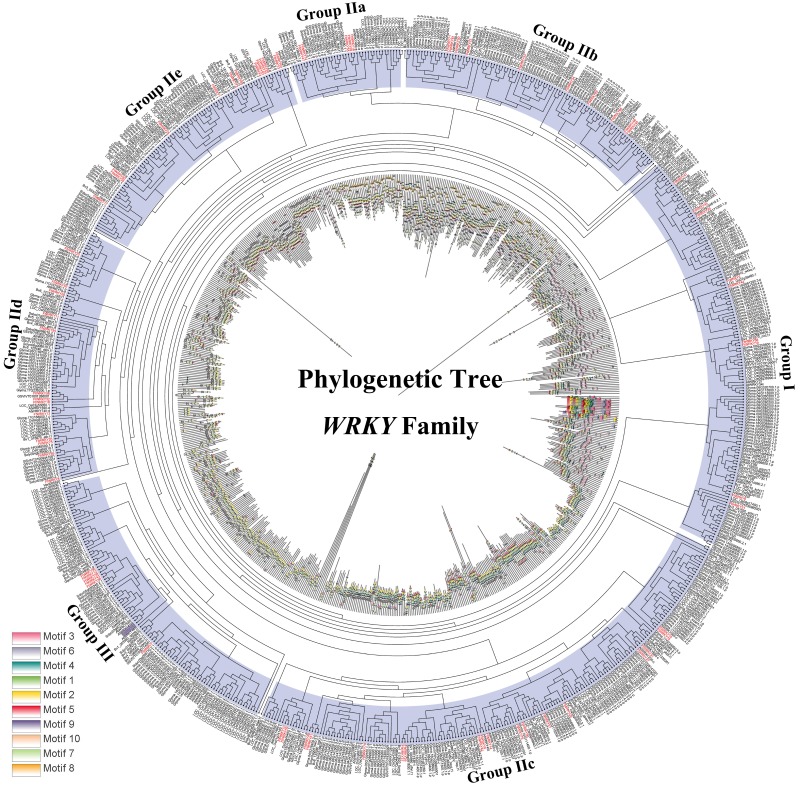
Phylogenetic relationships and motif compositions of WRKY proteins from seven different plant species. Outer layer: an unrooted phylogenetic tree constructed with the ML method. Inner layer: distribution of conserved motifs in WRKY proteins. The different-colored boxes represent the different motifs and their positions in the WRKY protein sequences.

**Figure 6:**
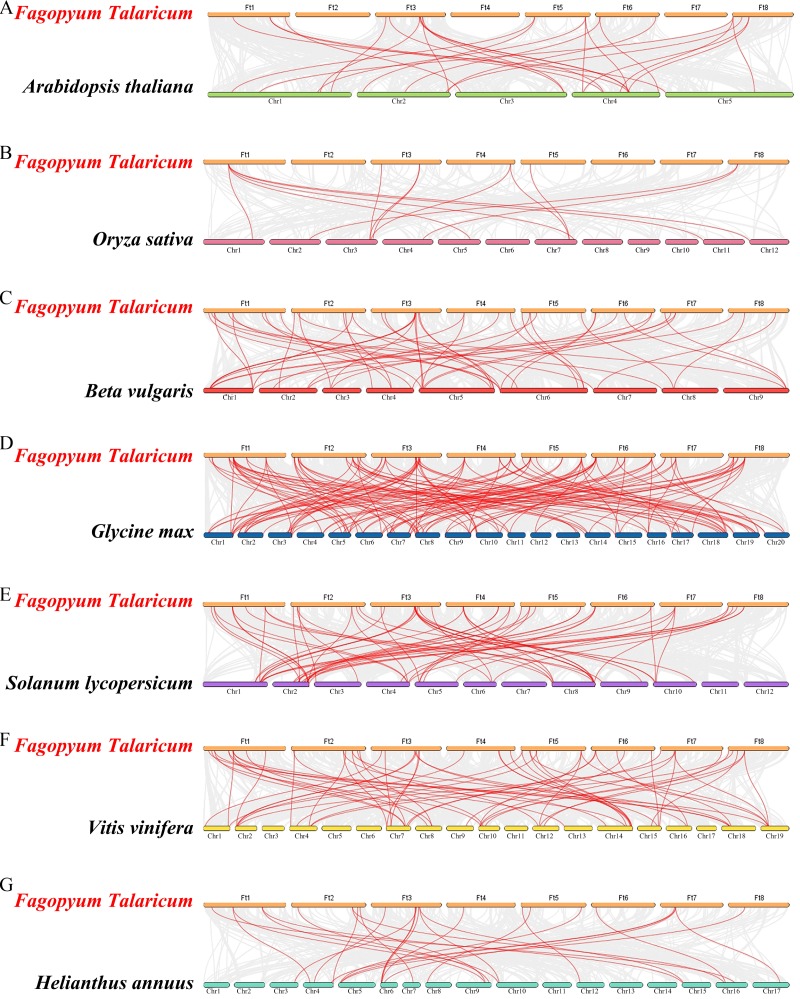
Synteny analysis of the WRKY genes in tartary buckwheat and seven representative plant species. The gray lines in the background indicate the collinear blocks within tartary buckwheat and other plant genomes; the red lines indicate the syntenic *WRKY* gene pairs. A–G represent the synteny relationship between *WRKY* genes in tartary buckwheat and that in *Arabidopsis thaliana*, *Oryza sativa*, *Beta vulgaris*, *Glycine max*, *Solanum lycopersicum*, *Vitis vinifera* and *Helianthus annuus*, respectively.

Differed from He et al.’s article, we further explore the evolutionary relationship of the WRKY family in multiple species. We set a phylogenetic tree with 76 *FtWRKY* genes and 72 *WRKY* genes in *A. thaliana*, 49 *WRKY* genes in *Beta vulgaris* (*B. vulgaris*), 296 *WRKY* genes in *G. max*, 59 *WRKY* genes in *Vitis vinifera (V. vinifera)*, 128 *WRKY* genes in *Oryza sativa* (*O. sativa*), 81 *WRKY* genes in *Solanum lycopersicum* (*S. lycopersicum*) and 97 *WRKY* genes in *Helianthus annuus* (*H. annuus*) (Fig. 5). From the phylogenetic tree, it can be seen that *WRKY* members in *H. annuus* were only grouped into Group IIb to IIe and Group III, *WRKY* members in other plants were grouped into Group I, Group IIa to IIe and Group III. With the MEME web server, we studied conserved motifs that were shared among WRKY proteins, and almost all WRKY proteins contained motifs 1 and 2. The motif composition of the WRKY protein was different in each subgroup, but the closer the evolution was, the more similar the motif composition was. For forecasting the syntenic relationship between Tartary buckwheat and other species, we set up the above seven representative species syntenic graphs (Fig. 6). *FtWRKY* genes were homologous to members in other species, and syntenic conservation was exhibited among *G. max* (125 orthologous gene pairs), *V. vinifera* (60 orthologous gene pairs), *S. lycopersicum* (60 orthologous gene pairs), *B. vulgaris* (70 orthologous gene pairs), *H. annuus* (27 orthologous gene pairs), *A. thaliana* (26 orthologous gene pairs) and *O. sativa* (13 orthologous gene pairs) (Fig. 6). Some *FtWRKY* genes, especially Tartary buckwheat and *G. max WRKY* genes, which might play a vital role in the evolution of the *WRKY* TF family, were related to three syntenic gene pairs at least (for example, *FtWRKY7/27/30*) (Table S3).

### Expression patterns of *FtWRKY* genes in Tartary buckwheat organs

**Figure 7:**
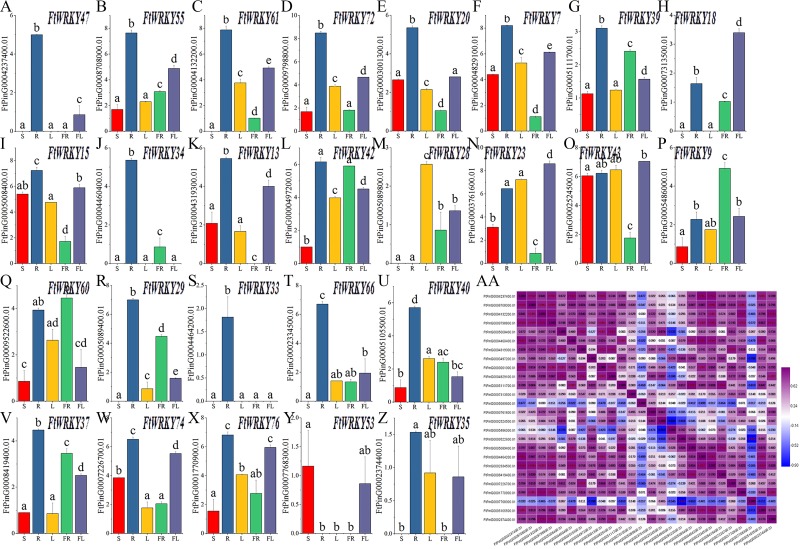
Tissue-specific gene expression of 26 tartary buckwheat *WRKY* genes and and the correlation between the gene expression patterns of *FtWRKY* genes. The expression patterns of 26 *FtWRKY* genes in flower, leaf, root, stem and fruit tissues were examined by qPCR (A–Z). Error bars were obtained from three measurements. The small letter(s) above the bars indicate significant differences (*α* = 0.05, LSD) among the treatments. AA shows the correlation between the gene expression patterns of *FtWRKY* genes. Purple: positively correlated; blue: negatively correlated. The red numbers indicate significant correlation at the 0.05 level.

Differed from He et al.’s article, our goal is to mine the *FtWRKY* genes that regulate growth and development. We chose 26 *WRKY* members from Groups I, II and III; that were homologous to *AtWRKY* genes and determined their expression patterns in leaves, roots, stems, flowers and fruits. The expression patterns of 26 *FtWRKY* genes were different in different tissues in these histograms. Except for *FtWRKY47*, *FtWRKY61*, *FtWRKY18*, *FtWRKY34*, *FtWRKY13*, *FtWRKY28*, *FtWRKY29*, *FtWRKY33*, *FtWRKY66*, *FtWRKY53* and *FtWRKY35*, all other genes were expressed in all tissues (Fig. 7A). Among them, *FtWRKY33* was only expressed in roots, *FtWRKY29*, *FtWRKY61* and *FtWRKY66* had no expression in stems, and *FtWRKY13* had no expression in fruits. The expression of 19 *FtWRKY* genes was the highest in roots, *FtWRKY53* was the highest in stems, *FtWRKY28* was the highest in leaves, *FtWRKY9* and *FtWRKY60* was the highest in fruits, and *FtWRKY18*, *FtWRKY23* and *FtWRKY43* were the highest in flowers (Fig. 7A).

Meanwhile, we analyzed the correlation of expression of different *FtWRKY* genes in roots, stems, leaves, flowers and fruits (Fig. 7B). Figure 7B shows that the expression of many genes was positively correlated, among which the expression patterns of *FtWRKY47* and 7 *FtWRKY* genes (*FtWRKY40, FtWRKY66, FtWRKY33, FtWRKY20, FtWRKY34, FtWRKY72* and *FtWRKY55)* were significantly positively correlated. By analyzing the correlation between the expression patterns of these genes in different tissues, the genes with highly correlated expression levels can be found, and the genes that may co-regulate plant growth and development can be preliminarily screened out.

### Differential expression of *FtWRKY* genes during fruit development in Tartary buckwheat

**Figure 8:**
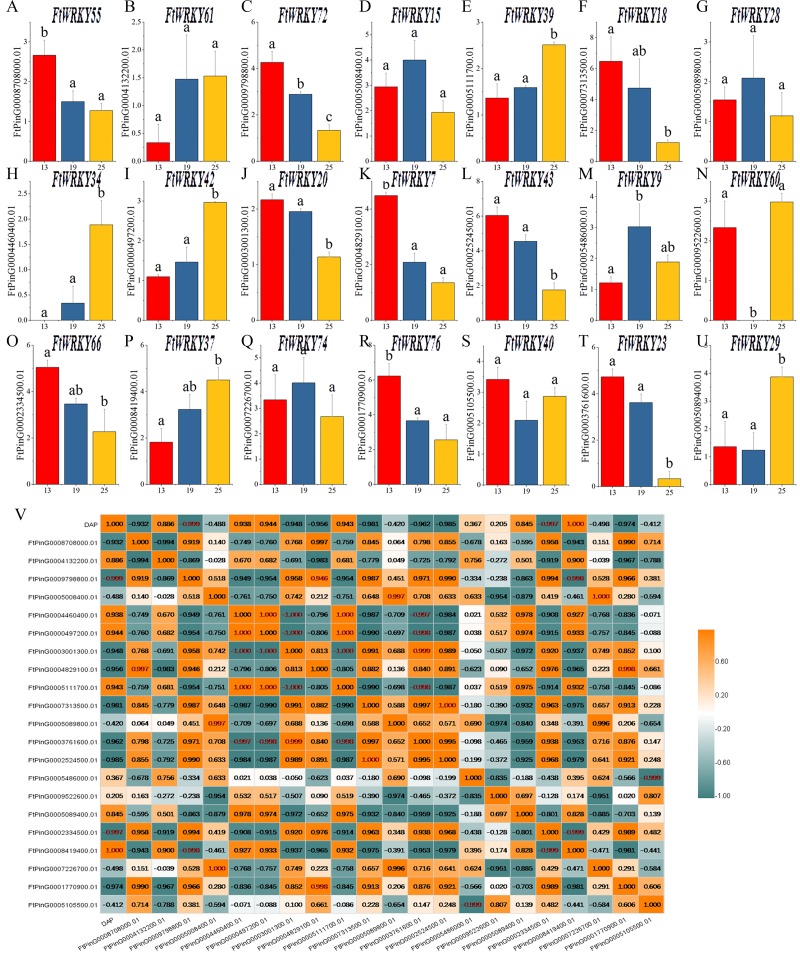
Gene expression of 21 tartary buckwheat WRKY genes during fruit development and the correlation between the gene expression of *FtWRKY* genes during fruit development. Expression patterns of 21 *FtWRKY* genes at the green fruit stage, the discoloration stage and the initial maturity stage examined by qPCR (A–U). Error bars were obtained from three measurements. The small letter(s) above the bars indicate significant differences (*α* = 0.05, LSD) among the treatments. (V) shows the correlation between the gene expression of *FtWRKY* genes during fruit development. Yellow: positively correlated; Dark green: negatively correlated. The red numbers indicate significant correlation at the 0.05 level.

WRKY family members play an important role in plant development and biological stress, but there are relatively few functional studies in fruit development. Tartary buckwheat is a kind of medicine and food crop, and its fruit contains abundant nutrients. Different from the focus of He et al.’s article, we also pay special attention to the expression of *FtWRKY* genes in fruits at different developmental stages. Of the 26 *FtWRKY* genes homologous to *AtWRKY* genes, 5 *FtWRKY* genes were not expressed in fruit (Fig. 7), so we explored the expression of the remaining 21 *FtWRKY* genes in different developmental stages of fruit. The expression levels of 21 *FtWRKY* genes at various developing periods of Tartary buckwheat fruits (green fruit stage, 13 DAP, discoloration stage, 19 DAP and initial maturity stage, 25 DAP) were measured using qRT-PCR. These histograms suggested that the abundance of *FtWRKY* gene transcripts differed significantly at different developing processes of fruit, showing that the *FtWRKY* genes had multiple effects during the developing stages of Tartary buckwheat fruit (Fig. 8A). *FtWRKY34* was not expressed at 13 DAP, *FtWRKY60* was not expressed at 19 DAP, and other genes were expressed at three developing stages (Fig. 8A). The expression levels of 9 *FtWRKY* genes (*FtWRKY55, FtWRKY72, FtWRKY18, FtWRKY20, FtWRKY7, FtWRKY43, FtWRKY66, FtWRKY76* and *FtWRKY23*) decreased gradually with the development of fruits, and 6 *FtWRKY* genes (*FtWRKY61, FtWRKY39, FtWRKY34, FtWRKY42, FtWRKY60* and *FtWRKY37)* increased gradually with the development of fruits, 4 *FtWRKY* genes (*FtWRKY* 15, *FtWRKY* 28, *FtWRKY* 9 and *FtWRKY* 74) had the highest expression in 19 DAP, and 2 *FtWRKY* genes (*FtWRKY* 40 and *FtWRKY* 29) had the lowest expression in 19 DAP.

During fruit development, there might be many genes involved in regulating this process together. We analyzed the correlation between their expression levels to preliminarily screen out the genes jointly expressed in fruit development, laying a foundation for research on the regulation of fruit development. The expression of *FtWRKY34* during different fruit developing periods was significantly negatively correlated with the expression patterns of *FtWRKY23* and *FtWRKY20*, while the expression levels of *FtWRKY34* were positively correlated with the expression levels of *FtWRKY39* and *FtWRKY42* (Fig.  8B). These genes may be jointly involved in the regulation of fruit development, which requires further experimental verification in the future.

## Discussion

### Evolution analysis of Tartary buckwheat WRKY gene family

The WRKY TF family is a large family that plays vital roles in many physiological processes and in adaptation to the environment (Du & Chen, 2010; Johnson, Ben & Smyth, 2002; Marie & Matton, 2004; Ying & Ulrike, 2007). He et al. (2019) identified 78 *FtWRKY* genes in the Tartary buckwheat genome, and 76 *FtWRKY* genes were identified in this study. By comparing the gene sequences identified in the two studies, we found that two more genes were identified in He et al.’s article, including *FtPinG0006884300.01* and *FtPinG0001732900.01* (Table S1). In this study, after *FtWRKY* genes were identified, SMART, Pfam and InterPro were used to confirm whether these *FtWRKY* genes had a WRKY conserved domain. It was found that *FtPinG0006884300.01* had no WRKY conserved domain; therefore, we removed this gene. While *FtPinG0001732900.01* showed two alternative splicings of messenger RNA, we only retained the longest transcript. Therefore, we identified 76 *FtWRKY* genes. Studies have shown that tandem duplication and segmental duplication events were one of reasons for gene family expansion (Cannon et al., 2004). He et al. (2019) identified two pairs of tandem duplication genes from *FtWRKY* genes, and the identification standard was that the chromosome region less than 100 kb contained more than two genes with more than 40% similarity as tandem duplication genes. However, in this study, we defined tandem duplicated genes as homologous genes with more than 70% similarity within 200 kb on a single chromosome, and no tandem duplication events were identified in *FtWRKY* genes, but 23 pairs of segmental duplication genes were found (Fig. 4). It has been reported that there are more *WRKY* genes in rice because the WRKY gene of Group III in rice has undergone tandem and segmental duplication events in evolution, while there are 13 pairs of tandem-duplicated gene pairs in 81 *WRKY* genes identified in tomato, which come from Group IIe (Huang et al., 2012; Wu, 2005). Different from other plants, the segmental-duplicated gene pairs in Tartary buckwheat were from Group I, Group IIb to IIe and Group III, and the segmental-duplicated gene pairs in Group II were the most abundant, which may be due to the large amount of *FtWRKY* members in Group II. In the course of evolution, duplicated genes may experience several different situations. The first is non-functionalization through silencing, the second is neofunctionalization through acquisition of novel function, and the third is that the duplicate genes are subfunctionalized to divide the original functions of the ancestor gene (Duarte, 2006; Lynch, 2000; Otto & Yong, 2002). *FtWRKY13* and *FtWRKY18* are a pair of segmental-duplicated genes (Fig. 4). *FtWRKY13* is not expressed in fruits but is highest in roots, while *FtWRKY18* is not expressed in stems and leaves but highest in flowers (Fig. 7). These genes both contain two introns, and analysis of their motif composition revealed that *FtWRKY18* contained motif 10, while *FtWRKY13* did not (Fig. 2). Further analysis of cis-acting elements in their promoters revealed that *FtWRKY13* contained GA, MeJA and auxin responsiveness elements, while *FtWRKY18* did not (Fig. 2). *FtWRKY39* and *FtWRKY60* are another pair of segmental-duplicated genes (Fig. 4). These genes are expressed in all tissues, but *FtWRKY39* is the most highly expressed in roots, while *FtWRKY60* is the most highly expressed in fruits (Fig. 7). *FtWRKY39* contains three introns, while *FtWRKY60* has only one intron. Analysis of their motif composition found that *FtWRKY39* contains motifs 4 and 9, while *FtWRKY60* did not (Fig. 2). Similarly, analysis of cis-acting elements in their promoters revealed that *FtWRKY60* contained wound responsiveness elements not found in *FtWRKY39* (Fig. 2). Therefore, we hypothesized that differences in motif composition and promoter cis-acting elements might lead to different expression patterns of these segmental-duplicated genes.

The structure of the phylogenetic tree according to an alignment of the WRKY domains of Tartary buckwheat and *A. thaliana* suggested that the 76 *FtWRKY* genes can be classified into three main Groups (I, II and III), which is consistent with the classification of *FtWRKY* genes by He et al. (2019). Meanwhile, multiple sequence alignment in this study indicated that the WRKY domains of two *FtWRKY* genes (*FtWRKY15* and *FtWRKY75*) in Group II c showed sequence variation (WRKYGQK was replaced by WRKYGKK); the WRKY domain of *FtWRKY49* in Group II b also displayed sequence variation (WRKYGQK was replaced by WRKYDQK) (Fig. S1). This phenomenon has also been observed in other plants, including *Glycine max*, wheat, *Camellia sinensis*, apple, and *Capsicum annuum* L (Diao et al., 2016; Meng et al., 2016; Sezer, Ebru & Turgay, 2014; Wu et al., 2016; Yu et al., 2016b). The W box is necessary for the specific binding of most WRKY TFs. Changes in the structure of WRKY affect the specific binding of TF to the W box; therefore, the three WRKY proteins may be meaningful to investigations examining their functions and binding specificities in great depth (Verk et al., 2008; Zhou et al., 2010).

Studies have shown that the diversity of exon-intron structure of genes is related to the evolution of genes (Shiu & Bleecker, 2003). The degree of intron-exon pattern differences between plant species closely reflects the evolutionary relationships of these species. A total of 44 *FtWRKY* genes have 2 introns in Tartary buckwheat, which come from Group IIa to IIe and Group III (Fig. 2). However, 42 of the *FtWRKY* genes identified by He et al. (2019) contain 2 introns, which is almost consistent with our results (He et al., 2019; Huang et al., 2016). Most *WRKY* members in other species also have 2 introns, 42 of the 85 *WRKY* genes in cassava have two introns, 30 of the 58 *WRKY* genes in physic nut have two introns, and 33 of the 71 *WRKY* genes in sesame have two introns (Li et al., 2017; Wei et al., 2016b; Xiong et al., 2013). Most *WRKY* genes from different plants have the same number of introns, showing that these *WRKY* members have similar complexity and relatively conserved structures.

### Diverse expression patterns of *FtWRKY* genes in Tartary buckwheat organs

Some valuable information concerning the functions of *FWRKY* genes that act a vital part in specific physiological processes in Tartary buckwheat was obtained by studying the expression patterns of the *FtWRKY* genes*.* We measured the expression of 26 *FtWRKY* genes in different Tartary buckwheat tissues. The expression levels of *FtWRKY40*, *FtWRKY66*, *FtWRKY33* and *FtWRKY34* were significantly positively correlated, both of which had the highest expression in roots, and they all belonged to Group II (Fig. 7). The results showed that many genes can be grouped according to their abundant expression in specific organs, a finding that might reflect their participation in the common metabolic and/or developmental processes. Trace elements are important constituents of certain enzymes, vitamins and growth hormones and have specific effects on the plant pigment system and on photosynthesis, respiratory metabolism, protein and nucleic acid metabolism, plant growth and hormones, and the primary function of lateral root and root hairs of plants is to absorb water and these trace elements (Shen, 2010). Studies have shown that *AtWRKY75* in *A. thaliana* can negatively regulate the formation of lateral and hairy roots (Devaiah, Karthikeyan & Raghothama, 2007). The homologous gene *FtWRKY42* of *AtWRKY75* was highly expressed in different tissues and the highest level in roots; therefore, the specific function of *FtWRKY42* was worthy of further study in the future (Fig. 7). Most of the *FtWRKY* genes (19 members) had the highest expression levels in roots, which is also observed in other plants, including grape, cassava, and cucumber (Ling et al., 2011; Wang et al., 2014; Wei et al., 2016b). These *FtWRKY* genes had the highest expression level in roots, indicating that they may play a crucial role in root growth and development. Among all the *FtWRKY* genes, *FtWRKY9/42* in Group IIc and *FtWRKY60* in Group I had high expression in fruits, while *FtWRKY42* expression gradually increased with fruit development, showing that *FtWRKY42* may be involved in fruit development and maturation (Figs. 7 and 8). *FtWRKY18*, *FtWRKY* 23 and *FtWRKY43* were highly expressed in flowers, suggesting that they may be related to flower development and flowering time (Fig. 7). *FtWRKY23* in Group IIb is also highly expressed in leaves, suggesting that it may also be involved in leaf development (Fig. 7). *FtWRKY43* in group III is highly expressed in stems, suggesting that it may be related to stem development (Fig. 7). WRKY genes with high expression usually play vital roles in plant development (Cheng et al., 2016). High expression levels of *FtWRKY43*, *FtWRKY15*, *FtWRKY7*, *FtWRKY23* and *FtWRKY42* in the four tissues indicated that these genes may play a vital role during plant growth and development. However, the functions of these *WRKY* family members need to be confirmed through a series of experiments in the future.

## Conclusions

The results of this study, such as gene identification, gene structure, motif composition, and chromosome location, were compared with the results reported by He et al. Meanwhile, through the comprehensive analysis of the FtWRKY gene family, candidate genes for regulating the growing and developing process of Tartary buckwheat could be preliminarily screened. Further study on the expression levels of the genes in different organs and fruits at different developing stages preliminarily verified the functions of these genes. This study provided a useful basis for further research studying the regulatory effect of *FtWRKY* genes on the growth and development of the Tartary buckwheat.

##  Supplemental Information

10.7717/peerj.8727/supp-1 Table S1 List of the 76 FtWRKY genes identified in this study

10.7717/peerj.8727/supp-2 Table S2 Analysis and distribution of conserved motifs in *Tartary Buckwheat* WRKY proteins

10.7717/peerj.8727/supp-3 Table S3 One-to-one orthologous relationships between *Tartary Buckwheat* and other plants

10.7717/peerj.8727/supp-4 Table S4 The primer sequences for qRT-PCR

10.7717/peerj.8727/supp-5 Figure S1 Alignment of the amino acid sequences of multiple FtWRKY and selected WRKY domains**.**

10.7717/peerj.8727/supp-6 Figure S2 Unrooted phylogenetic tree representing relationships among the WRKY genes of tartary buckwheat and *A. thaliana* use NJ method The genes in tartary buckwheat are marked in red, while those in *A. thaliana* are marked in black. The different-colored arcs indicate different groups (or subgroups) of WRKY genes.

10.7717/peerj.8727/supp-7 Supplemental Information 1 Raw data

## Abbreviations

*A. thaliana*: Arabidopsis thaliana
*FtWRKY*: Fagopyrum tataricum WRKY
PI: isoelectric point
GSDS: Gene Structure Display Server
HMM: Hidden Markov Model
MCScanX: Multiple collinear scanning toolkits
NJ: Neighbor-joining
qRT-PCR: quantitative real-time polymerase chain reaction
TBGP: *Tartary Buckwheat* Genome Project

## Additional Information and Declarations

### Data Availability

The following information was supplied regarding data availability: The raw data is available as a Supplementary File.
