# Supplementary figures and images for: Genome-wide investigation of WRKY transcription factors in Tartary buckwheat (Fagopyrum tataricum) and their potential roles in regulating growth and development
Source: PeerJ. 2020 Mar 5;8:e8727. doi: 10.7717/peerj.8727 (PMC7060923; doi:10.7717/peerj.8727)

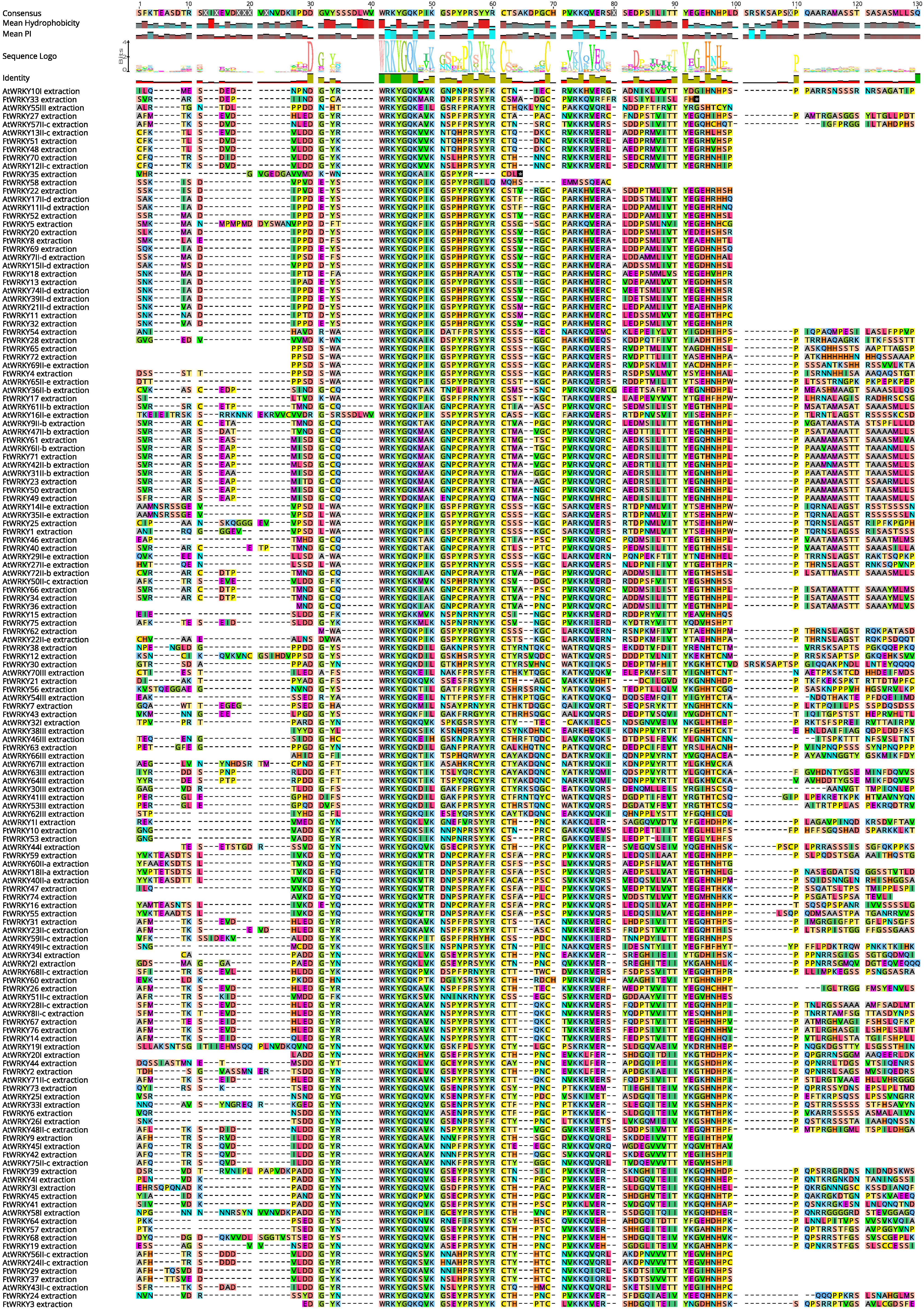

Supplement: Figure S1 [file peerj-08-8727-s005.jpg]

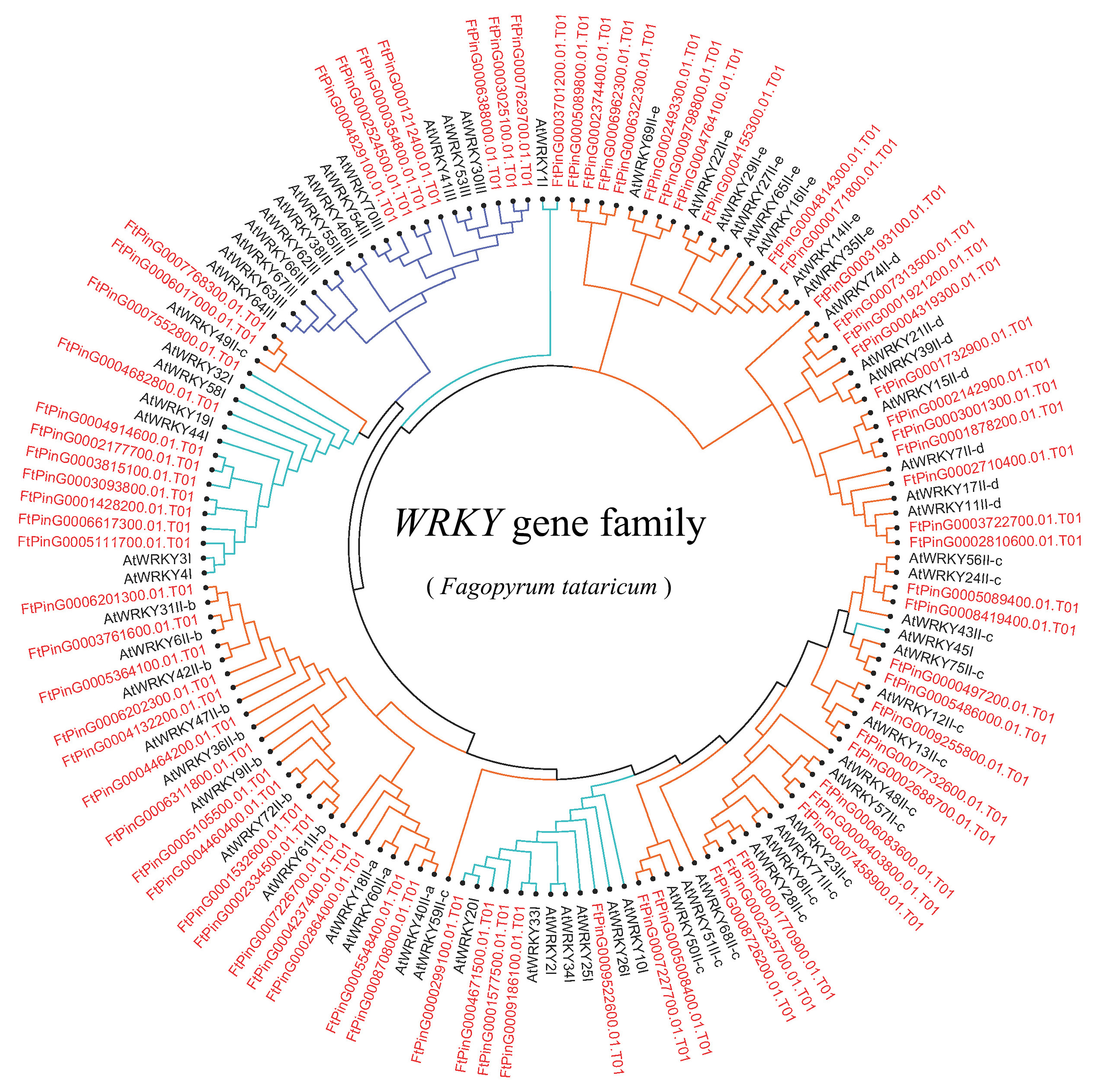

Supplement: Figure S2 — The genes in tartary buckwheat are marked in red, while those in A. thaliana are marked in black. The different-colored arcs indicate different groups (or subgroups) of WRKY genes. [file peerj-08-8727-s006.jpg]
